# Hematology profile analysis in coronavirus disease 2019 (COVID-19) patients

**DOI:** 10.1515/almed-2022-0053

**Published:** 2022-10-13

**Authors:** Felisia Setio, Darwati Muhadi, Asvin Nurulita, Mansyur Arif, Irawaty Djaharuddin, Arifin Seweng

**Affiliations:** Hasanuddin University, Makassar, Indonesia; Dr. Wahidin Sudirohusodo Hospital, Makassar, Indonesia

**Keywords:** comorbidity, COVID-19, Ct value, hematology profile, inflammation, severity

## Abstract

**Objectives:**

Some hematological parameters were reported as markers to assess severity of COVID-19 patients. Comorbidities were risk factors for severe COVID-19. Differences in hematology profile based on severity and comorbidity, and correlation between hematology profile and Ct value were never studied at Makassar, Indonesia. The aim of this study were to know the differences of hematology profile based on severity and comorbidity, and the correlation between hematology profile and Ct value in COVID-19 patients.

**Methods:**

This study was retrospective, cross-sectional of confirmed COVID-19 patients who had been hospitalized at Dr. Wahidin Sudirohusodo hospital, Makassar, since June to August 2020. Hematology profile, Ct value, comorbidity, and severity of COVID-19 patients were obtained from Hospital Information System Data.

**Results:**

From 217 patients, subjects were 102 (47%) male dan 115 (53%) female, 127 mild-moderate patients (58.5%) and 90 severe patients (41.5%), 143 patients (65%) without comorbidity, 74 patients (35%) with comorbidity. White blood cells (WBC), red cell distribution width (RDW), neutrophil and monocyte count, and neutrophil lymphocyte ratio (NLR) were significantly higher in severe patients than mild-moderate patients (p<0.05), besides RBC, hemoglobin, hematocrit, lymphocyte and thrombocyte count were significantly lower in severe patients than mild-moderate patients (p<0.05). Hematology profile was not different significantly based on comorbidity and was not correlated significantly with Ct value, except eosinophil count (r=0.161; p=0.018).

**Conclusions:**

We suggest that hematology profile could predict the severity of COVID-19 patients. Moreover, eosinophil count could be considered to predict the infectivity of patient with COVID-19.

## Introduction

Coronavirus disease 2019 (COVID-19) pandemic, which caused by virus named severe acute respiratory syndrome coronavirus-2 (SARS-CoV-2) and originated from Wuhan, China, has caused 36,754,395 confirmed cases and 1,064,838 deaths around the world based on the World Health Organization (WHO) data at October 11, 2020, including in Indonesia [1]. Confirmed COVID-19 cases in Indonesia were recorded to 328,952 patients until October 11, 2020, with 11,765 death cases (3.6%). South Sulawesi district is the fifth highest COVID-19 cases after Jakarta (capital district), East Java, West Java, Middle Java district, with total 16,399 confirmed cases and 431 deaths (2.63%) [2].

SARS-CoV-2 enters human cell through binding of its spike protein and ACE2 receptor which is expressed in multiple organ such as lung, heart, kidney, and intestinal and vascular endothelium. The clinical manifestation and severity of COVID-19 can vary greatly. Most COVID-19 cases are asymptomatic or had mild to moderate symptoms, usually manifest as flu like symptoms or mild pneumonia [3], [4], [5]. However, around 14% of patients have severe disease and 5% are critical with respiratory failure, sepsis, or multi-organ dysfunction [3], [4], [5], [6]. Patients with comorbidity, such as hypertension, diabetes mellitus (DM), cardiovascular disease, chronic renal disease, and chronic respiratory disease had higher risk at severe COVID-19 condition [3, 7, 8].

Several laboratory test are routinely assessed in COVID-19 patients in our district, some of them are complete blood count (CBC) and molecular analysis. Hematological profiles in COVID-19 patients have been studied, and several parameters have been known to provide clinical value in COVID-19 infection. Severe COVID-19 patients tend to have leukocytosis, neutrophilia, lymphopenia, eosinopenia, and thrombocytopenia. Hemoglobin (Hb) in severe COVID-19 was lower than mild–moderate patients although it was still in normal range [9], [10], [11]. Neutrophil lymphocyte ratio (NLR) was reported higher in COVID-19 positive patients than COVID-19 negative patients [4]. NLR was also suggested as a predictor of poor outcome in COVID-19 patients [4, 12]. COVID-19 patients with high NLR were reported to have increased inflammatory markers such as interleukin-6 and tumor necrosis factor-alpha (TNF-α). Moreover, red cell distribution width (RDW), marker of anisocytosis in hemogram, has been associated with recurrent hospitalizations in patients with COVID-19 [13]. Several hematological parameters also associated with specific comorbidity such as DM and hypertension [14, 15]. Some hematology profile was associated with predictors of frailty in diabetics during COVID-19 [16]. Therefore, it can be assumed that inflammatory hemogram indices could be associated with COVID-19 infection.

Other laboratory test that is routinely assessed is reverse transcription-polymerase chain reaction (RT-PCR). Cycle threshold (Ct) value was obtained from RT-PCR, and had negative correlation with viral load. The correlation between Ct value and patient’s severity condition was still debateful and understudied [17], [18], [19], [20]. The differences in Ct value between COVID-19 patients with and without comorbidity had also not been reported.

In this study, we try to analyze the clinical value of these two routinely assessed laboratory tests and try to describe the hematology profile of COVID-19 patients based on the severity and comorbidity, and also the correlation between hematology profile and Ct values of confirmed COVID-19 patients.

## Materials and methods

This study was retrospective, cross-sectional of confirmed COVID-19 patients aged ≥18 years old based on RT-PCR results who had been hospitalized at Dr. Wahidin Sudirohusodo hospital, Makassar, South Sulawesi district, Indonesia, since June to August 2020 using data from Hospital Information System Data. After deidentification process by removing the personal information (name and ID), demographic data (age, sex, and comorbidity), CBC results and Ct values from RT-PCR were extracted from medical record at the first and same time when the COVID-19 patients assessed in emergency department. Patients with incomplete medical record and cancer were excluded from this study.

Hematology profile, based on CBC results which have been collected in this study were hemoglobin, hematocrit, red blood cell (RBC) count, red cell distribution width (RDW), white blood cell (WBC) count, absolute eosinophil count, absolute basophil count, absolute neutrophil count, absolute lymphocyte count, absolute monocyte count, and thrombocyte count which obtained from Sysmex XN-1000 an automated hematology analyzer (Sysmex Corporation, Kobe, Japan). NLR was obtained from the calculation of the absolute neutrophil count divided by absolute lymphocyte count. The severity of COVID-19 in this study was divided into 2 groups, namely mild–moderate and severe groups. The severity of COVID-19, adopted from Indonesian Guidelines for Prevention and Control of COVID-19 (5th-Revision), were assessed by pulmonologist in emergency department, then they will determine the type of inpatient ward, either in general infection ward or intensive care infection ward. Mild–moderate COVID-19 patients with no signs and symptoms of severe pneumonia were hospitalized in general infection ward, whereas severe COVID-19 patients with severe pneumonia (respiratory distress >30 breath/minute or oxygen saturation <90%) were treated in intensive care infection ward. COVID-19 patients were confirmed based on the detection of positive SARS-CoV-2 RNA by testing the ORF1ab and/or *N* gene (Sansure Biotech Inc, Hunan, China) from nasopharyngeal swab specimens using the RT-PCR method (Lightcycler^®^ 480 II, Roche Diagnostics, Mannheim, Germany), examined by the Clinical Pathology Laboratory at Dr. Wahidin Sudirohusodo Hospital, Makassar City, Indonesia. Confirmed COVID-19 patients without symptoms were not hospitalized, so they were not included in this study. Based on the presence of comorbidity, COVID-19 patients were divided into 2 groups, namely: COVID-19 without comorbidity if there was no comorbidity and with comorbidity if they had 1 or more comorbidities.

The data were processed using IBM SPSS Statistics (version 22.0, IBM Corp., Armonk, NY, USA). Normality analysis was performed using Kolmogorov-Smirnov test. The independent t-test statistical test was used to compare the mean of hematology parameters and the Ct values based on the degree of disease severity and the presence of comorbidity, while the Pearson’s correlation test was used to assess the correlation between hematology parameters and Ct values in confirmed COVID-19 patients. The statistical test results are significant if p-value <0.05. Research permission was obtained from the Health Research Ethics Commission, Hasanuddin University Medical Faculty – dr. Wahidin Sudirohusodo Hospital, Makassar, and Hasanuddin University Hospital, Makassar, with number 518/UN4.6.4.5.31/PP36/2020.

## Results and discussion

Patients aged ≥18 years with confirmed COVID-19 who were hospitalized at Dr. Wahidin Sudirohusodo hospital from the period June 2020 - August 2020 and had met the inclusion criteria were totally 221 patients, 4 patients with cancer were excluded, so that the subjects of this study were 217 people. Table 1 provides an overview of the demographic characteristics of the patients in this study.

**Table 1:** Demographic characteristics of the COVID-19 patients.

Criteria	Number (%)
**Sex**	
Male	102 (47%)
Female	115 (53%)

**Age** (18–85 years) Mean (SD)=41.8 (15.3)	
>18–30 years	73 (34%)
31–45 years	59 (27%)
46–59 years	51 (24%)
≥60 years	34 (15%)

Mild-moderate	127 (58.5%)
Severe	90 (41.5%)
Without comorbidity	143 (65%)
With comorbidity	74 (35%)
Hypertension	40 (18%)
Diabetes mellitus	27 (12%)
Cardiovascular disease	15 (7%)
Chronic kidney disease	13 (6%)
Cerebrovascular disease	3 (1%)
HIV-AIDS	3 (1%)
Others	11 (5%)

	Minimum – maximum	Mean (SD)
WBC, cell/µL	2,700–32,400	9,563.13 (4,405.96)
RBC, ×10^6^ cell/µL	2.00–6.63	4.47 (0.86)
Hb, g/dL	5.8–19.1	1.73 (2.53)
HCT, %	17–56	37.50 (7.20)
PLT, ×10^3^ cell/µL	48–651	307.59 (108.98)
RDW, %	11.4–24.0	13.77 (1.97)
Neutrophil, cell/µL	1,504–29,808	6,645.29 (4,399.95)
Lymphocyte, cell/µL	280–5,947	2,007.99 (950.55)
Monocyte, cell/µL	143–5,798	684.15 (446.69)
Eosinophil, cell/µL	0–1,184	178.57 (176.39)
Basophil, cell/µL	0–202	40.36 (29.95)
NLR	0.72–41.87	5.08 (6.54)
Ct values	15.92–39.37	30.05 (5.39)

Table 2 illustrates the differences in hematology profiles based on mild-moderate groups and severe groups, as well as with and without comorbidity. WBC, RDW, the number of neutrophils, monocytes, NLR were significantly higher in patients with severe COVID-19 than mild-moderate groups (p<0.05), while RBC, hemoglobin, hematocrit, platelet count, lymphocytes were significantly lower in severe groups than mild-moderate COVID-19 patients (p<0.05). The number of eosinophils, and basophils did not differ significantly between severe and mild-moderate COVID-19 patients. Hematology profiles did not differ significantly in COVID-19 patients with and without comorbidity.

**Table 2:** Differences in hematology profiles in COVID-19 patients based on patient’s severity and presence of comorbidity.

CBC parameter	Patient’s severity			Presence of comorbidity		
	Mild-moderate (n=127)	Severe (n=90)	p-Value^a^	Without comorbidity (n=143)	With comorbidity (n=74)	p-Value^a^
	Mean (SD)	Mean (SD)		Mean (SD)	Mean (SD)	
WBC, cell/µL	8,148.82 (2,712.78)	11,558.89 (5,460.11)	<0.001	9,518.88 (4,618.72)	9,648.65 (3,991.54)	0.830
RBC, ×10^6^ cell/µL	4.83 (0.63)	3.96 (0.87)	<0.001	4.51 (0.84)	4.39 (0.89)	0.319
Hb, g/dL	13.67 (2.0)	11.39 (2.60)	<0.001	12.83 (2.52)	12.52 (2.54)	0.390
HCT, %	40.35 (5.32)	33.48 (7.60)	<0.001	37.78 (7.09)	36.97 (7.43)	0.444
PLT, ×10^3^ cell/µL	323.10 (100.10)	285.71 (117.52)	0.012	308.70 (100.60)	305.46 (124.29)	0.847
RDW, %	13.36 (1.87)	14.36 (1.97)	<0.001	13.73 (1.97)	13.85 (1.98)	0.658
Neutrophil, cell/µL	4,883.54 (2,421.12)	9,131.51 (5,292.09)	<0.001	6,559.48 (4,649.82)	6,811.35 (3,896.57)	0.674
Lymphocyte, cell/µL	2,415.43 (863.51)	1,433.20 (752.45)	<0.001	2,084.55 (970.92)	1,860.23 (897.81)	0.092
Monocyte, cell/µL	616.08 (227.18)	780.29 (628.70)	<0.001	648.13 (282.76)	753.86 (653.79)	0.187
Eosinophil, cell/µL	189.27 (135.78)	163.48 (221.40)	0.290	182.58 (181.72)	170.82 (166.54)	0.634
Basophil, cell/µL	42.93 (30.01)	36.94 (29.61)	0.147	41.99 (31.36)	37.47 (26.89)	0.270
NLR	2.36 (2.05)	8.93 (8.51)	<0.001	4.84 (6.56)	5.55 (6.53)	0.455

^a^Independent t test, p-value <0.05 was considered significant.

WBC, Hb, platelets, RDW, NLR, neutrophils, lymphocytes, monocytes, and basophils were not significantly correlated with Ct values ​​in COVID-19 patients. The number of eosinophils had a weak positive and significant correlation with Ct value (r=0.161, p=0.018) (Table 3).

**Table 3:** Correlation of hematology profiles with Ct value in COVID-19 patients.

Parameter Routine blood profiles	Ct values	
	r	p-Value^a^
WBC	0.036	0.599
Hb	−0.099	0.150
PLT	0.074	0.280
RDW	−0.041	0.549
NLR	0.016	0.810
Neutrophil	0.000	0.999
Lymphocyte	0.102	0.137
Monocyte	0.060	0.384
Eosinophil	0.161	0.018
Basophil	0.083	0.223

^a^Pearson’s correlation test, p-value <0.05 was considered significant.

There were no significant differences in Ct values in patients with mild-moderate COVID-19 and severe COVID-19 patients (p=0.526) and in COVID-19 patients with and without comorbidity (p=0.831) (Table 4).

**Table 4:** Differences in Ct value based on patient’s severity and presence of comorbidity.

	Patient’s severity			Presence of comorbidity		
	Mild-moderate (n=127) Mean (SD)	Severe (n=90) Mean (SD)	p-Value^a^	Without comorbidity (n=143) Mean (SD)	With comorbidity (n=74) Mean (SD)	p-Value^a^
Ct value	29.86 (5.34)	30.33 (5.48)	0.526	29.99 (5.49)	30.16 (5.24)	0.831

^a^Independent t test, p-value <0.05 was considered significant.

WHO and the Indonesian Guidelines for Prevention and Control of COVID-19 (5th-Revision) reported that 20% of patients were clinically severe to critically ill, while 80% of patients were clinically mild–moderate. Characteristics of the population of COVID-19 patients who were hospitalized at Dr. Wahidin Sudirohusodo Hospital based on the severity of the patient was different, namely 41.5% with severe groups and 58.5% with mild–moderate groups. The higher percentage of severe COVID-19 patients at Dr. Wahidin Sudirohusodo Hospital was caused by Dr. Wahidin Sudirohusodo hospital is one of the main referral center for COVID-19 patients in South Sulawesi district in Indonesia. The mean age of confirmed COVID-19 patients in this study was 41.8 ± 15.3 years, which is the productive age group. 65% of COVID-19 patients who are hospitalized at Dr. Wahidin Sudirohusodo Hospital were without comorbidity, while 35% of them had comorbidity with the three most common comorbidity were hypertension (18%), diabetes mellitus (12%), and cardiovascular disease (7%) [2, 3, 6].

The mean number of WBC, neutrophils, and monocytes in severe COVID-19 patients were higher than mild-moderate patients, while the lymphocyte count was significantly lower in severe COVID-19 patients. The higher WBC count in patients with severe COVID-19 can be caused by the possibility of bacterial co-infection in severe cases or a more severe immune process. These results were associated with the review study by Karthabil et al., that the WBC count is generally normal in COVID-19 patients, but in severe COVID-19 cases the WBC count is higher than mild-moderate patients. The lymphocyte count was lower, even lymphopenia (mean lymphocyte count in severe patients was 1,433.20 cells/µL) was found in patients with severe COVID-19 than mild-moderate patients, and these results are consistent with the results of other studies (Karthabil et al., Liu et al. Guan et al., Qin et al., Yang et al.). This was further explained by Yang et al. and Qin C et al. that in severe COVID-19 patients there was a decrease in regulatory T lymphocytes and the percentage of memory T cells and an increase in the percentage of naive T lymphocytes which indicated a dysfunction in the inflammatory response that was greater in severe condition, so that the severity of the organ failure is heavier. The study of Yang et al. and Gustine et al. explained that SARS-CoV-2 can directly infect lymphocytes and macrophages. Autopsy evaluation of COVID-19 patients with lymphopenia showed severe lymphocyte cell death and macrophages in lymph nodes and splenic, it is suspected that activated macrophages express inflammatory cytokines thereby inducing lymphocyte necrosis and apoptosis. The higher neutrophils count in severe cases of COVID-19 was explained by Yang et al. because the disrupted lymphocytes in COVID-19 patients make it easier to develop bacterial infections, thus triggering the activation and recruitment of neutrophils in the blood. The study of Karthabil et al., and Liu et al. also reported that the number of neutrophils is a predictor of disease severity [7, 9, 10, 21], [22], [23].

NLR is a parameter which combines neutrophilia and lymphophenia to more acurately predict systemic inflammation. Because it is a ratio, it is less affected to pre-analytic factor, and is easily obtainable from routine laboratory test. NLR is associated with several inflammatory conditions, such as thyroiditis, ulcerative collitis, uncontrolled diabetes mellitus, irritable bowel disease, and more recently, COVID-19 infection [4, 24], [25], [26], [27]. Moreover, NLR was consistently reported to be higher in severe COVID-19 patients, with a cut-off of ≥3.13 for the high risk category and <3.13 for the low risk category for the possibility of severe illness, which corresponding with this study where the mean NLR was obtained 8.93 in severe cases and 2.36 in mild–moderate cases. There was an association between increase of NLR with the increase of some inflammatory markers in patient with more severe COVID-19, such as C-reactive protein (CRP), interleukin-6, TNF-α, and serum ferritin [4, 7, 9, 10, 21], [22], [23, 28].

There were higher monocyte counts in severe cases than mild–moderate cases in this study. This is in contrary to the study of Yang et al. and Khartabil et al., that monocytes are lower in severe cases because the activated monocyte system can induce inflammatory cytokines that exacerbate lung and other tissue damage. The eosinophils and basophils count was lower in patients with severe COVID-19 although in this study they were not significant, this was due to the low concentrations of normal eosinophils and basophils in the blood, the researchers did not separate the critical ill COVID-19 patients with sepsis. Eosinopenia has been studied as a marker of sepsis [7, 9, 10, 21], [22], [23, 29]

Hemoglobin level was lower and RDW was higher in patients with severe COVID-19 cases, this suggests that a more severe inflammatory process interferes with the erythropoiesis process. The platelet count was generally still in the normal range in this study, but the number is significantly lower in patients with severe COVID-19 [7], [8], [9, 30], [31], [32].

The presence of comorbidity did not affect the hematology profile of COVID-19 patients in this study. Manson’s study supports these results, reported that COVID-19 patients with a hyperinflammatory condition on admission to hospital had fewer comorbidities than those without hyperinflammation. In contrast, the study of Zhou et al. Chrsitensen et al. stated that more comorbidities lead to a worse outcome. This is because this study compared hematology profiles based on the presence of comorbidity at the time of admission to hospital, but did not follow the dynamics of hematology profile in serial time until the patient went home or died [33], [34], [35].

The Ct value in this study was found to be not significantly different in the degree of disease severity. This result was in contrast with the research of Rao et al., which stated that the Ct value is an indirect method of calculating the number of coronavirus copies, and the Ct value had a negative correlation with the degree of disease severity. This study suggested that a low Ct value does not necessarily lead to a bad clinical impact. This result is supported by Argyropoulos study which stated that viral load titres were lower in confirmed COVID-19 patients who were hospitalized than outpatients, and also reflected the time of infection onset, while Singanayagam and Young et al. stated that the Ct value did not correlate with the degree of severity. Ct values did not differ significantly in COVID-19 patients based on comorbidity. This indicates that the presence of comorbidity does not affect a person’s infectivity [17, 20, 36].

The Ct value was not clinically significant with almost all hematology profiles, except for the number of eosinophils. These results reinforce that the patient’s clinical impact on poor COVID-19 was caused by a more inflammatory condition and dysregulation of the body’s immune system than the patient’s Ct value at the initial day of hospital admission. However, based on the results of this study, the eosinophil count can be used to help estimate the viral load besides the Ct value in COVID-19 patients even though the correlation is weak, which the lower the eosinophil count, the lower the Ct value, and the higher the level of infectivity, although it still requires further research with a larger sample size and is carried out using the cohort method. Another study from Li et al. stated that eosinophil count decreased significantly during initial diagnosis and the repositive episode than negative stage COVID-19. Moreover, eosinophil count can be improved when the viral load in patients is reduced [17, 18, 37].

This study further marks the importance of several hematology parameters in predicting the severity of COVID-19 patients to facilitate early and adequate treatment. Complete blood count is a simple, cost-effective, routinely tested, and available in most medical center, yet has significant clinical value. In the setting of COVID-19 screening, lower eosinophil level could predict lower Ct value which means that the patient could more potentially spread SARS-CoV-2. In confirmed COVID-19 patients, NLR could further used as an alternative parameter to assess inflammatory state.

Some limitations of this study are this study did not follow the hematology profiles and the patient’s Ct value in serial time during hospitalization, so that the dynamics of changes in body response that can be seen from changes in hematology profiles and dynamics of Ct values cannot be assessed, and we do not know the effect of comorbidity on patient outcome. The effect of comorbidity in this study was analyzed in general, so that the researcher could not know the effect of each comorbidity whether there was a more significant effect on the hematology profile in some certain comorbidities. Conditions from the comorbidity itself, such as chronic renal failure, HIV can affect the hematology profile of COVID-19 patients.

In conclusion, we suggest that hematology profile could predict the severity of COVID-19 patients. Moreover, eosinophil count could be considered to predict the infectivity of patient with COVID-19. Researchers suggest conducting further research with a cohort method on the dynamics of hematology profiles in COVID-19 patients.
